# Association between the Venous Excess Ultrasound (VExUS) score and acute kidney injury in critically ill patients with sepsis: a multicenter prospective observational study

**DOI:** 10.1186/s13613-025-01529-w

**Published:** 2025-07-23

**Authors:** Jia Song, Gongze Chen, Detian Lai, Li Zhong, Haozhe Fan, Weihang Hu, Minjia Wang, Caibao Hu, Wenwei Chen, Ziqiang Ming, Shijin Gong, Qiancheng Luo

**Affiliations:** 1 Department of Critical Care Medicine, Zhejiang Hospital, No. 12, Lingyin Road, Xihu District, Hangzhou, 310013 Zhejiang China; 2 Department of Critical Care Medicine, Shanghai Pudong New Area Gongli Hospital, No. 219, Miaopu Road, Pudong New Area, Shanghai, 200135 China; 3 Department of Critical Care Medicine, The No.1 Peoples Hospital of Huzhou, No. 158, Guangchang Hou Road, Wuxing District, Huzhou, 313000 Zhejiang China; 4 Department of Critical Care Medicine, Jinhua Municipal Central Hospital, No. 365, Renming Dong Road, Wucheng District, Jinhua, 321000 Zhejiang China; 5 The 2nd Clinical Medical College, Zhejiang Chinese Medical University, No. 548, Binwen Road, Binjiang District, Hangzhou, 310053 Zhejiang China; 6 Department of Critical Care Medicine, The People’s Hospital of Xinchang, No. 117, Gushan Zhong Road, Nanming District, Shaoxing, 312500 Zhejiang China

**Keywords:** ICU, Sepsis, Ultrasound, Venous congestion, VExUS, Acute kidney injury, Mortality

## Abstract

**Background:**

Venous congestion is associated with adverse clinical outcomes in critically ill patients, yet its assessment remains challenging. Recently, the Venous Excess Ultrasound (VExUS) score has shown great potential as a non-invasive tool for assessing venous congestion in cardiac patients. However, the relationship between VExUS and clinical outcomes in patients with sepsis remains understudied. This study aims to evaluate the incidence of venous congestion based on VExUS assessment within the first 5 days of intensive care unit (ICU) admission in critically ill patients with sepsis, and to investigate the association between VExUS and clinical outcomes.

**Methods:**

We conducted a prospective, observational study in four ICUs, enrolling adult patients with sepsis who stayed in the ICU for at least 24 h. VExUS assessments were performed on days 1 (within 24 h), 3 (48–72 h), and 5 (96–120 h) following ICU admission. Patients were classified according to VExUS score ≥ 2 or < 2. The primary outcome was the prevalence of acute kidney injury (AKI) during ICU stay, while secondary outcomes included 30-day mortality, ICU mortality, and requirement for renal replacement therapy (RRT).

**Results:**

Among the 108 patients included, 18% (19 patients) showed VExUS score ≥ 2 on day 1 of ICU admission, and the prevalence progressively decreased to 15% (15 patients) by day 3 and 6% (6 patients) by day 5. The VExUS score ≥ 2 was not associated with AKI (OR 1.82, 95% CI 0.62–5.31, *p* = 0.274), 30-day mortality (OR 0.82, 95% CI 0.28–2.4, *p* = 0.711), ICU mortality (OR 1.12, 95% CI 0.41–3.04, *p* = 0.82), or requirement for RRT (OR 2.29, 95% CI 0.68–7.64, *p* = 0.179). There was no significant correlation between VExUS and central venous pressure (coefficient: − 0.019, 95% CI −0.01 to 0.05, *p* = 0.204).

**Conclusion:**

In critically ill patients with sepsis, approximately 20% exhibit early (within 24 h of ICU admission) venous congestion, with the prevalence progressively decreasing over the subsequent 5 days. Venous congestion assessed by VExUS was not associated with the occurrence of AKI or with mortality.

*Trial registration*: Chinese Clinical Trial Registry, ChiCTR2200066987. Registered 22 December 2022, https://www.chictr.org.cn/

**Supplementary Information:**

The online version contains supplementary material available at 10.1186/s13613-025-01529-w.

## Background

Sepsis remains a major challenge to clinicians in intensive care unit (ICU), with high morbidity and mortality [1]. Fluid resuscitation is one of the cornerstones of sepsis management. However, fluid resuscitation is a double-edged sword: while it improves tissue perfusion, it also carries the potential risk of fluid overload [2, 3]. Several studies have suggested an association between fluid overload and an increased incidence of acute kidney injury (AKI) and mortality in patients with sepsis [4–6].

Recent studies have suggested that venous congestion is one of the potential pathophysiological mechanisms for the harmful effects of fluid overload on end-organ function [7, 8], prompting a greater attention to the assessment of venous congestion, despite certain clinical challenges. Clinically, several methods are available to evaluate venous congestion, each with its inherent limitations. These limitations highlight the need for a reliable, economical, and non-invasive method to assess venous congestion. Recently, Beaubien-Souligy and colleagues developed a novel “Venous Excess Ultrasound (VExUS)” scoring system in a post-cardiac surgery cohort [9]. This four-point ultrasound examination technique integrates the inferior vena cava (IVC) diameter analysis with the Doppler flow patterns of the hepatic, portal, and intrarenal venous to provide a quantifiable assessment of venous congestion, and showed an association with AKI development in post-cardiac surgery patients. This pioneering study has sparked great interest in using VExUS as a non-invasive method for evaluating venous congestion in different clinical settings. however, the results remain debated. The discrepancy in results between studies underscores the necessity for expanded validation before implementing VExUS in diverse patient cohorts, such as those with sepsis, acute respiratory distress syndrome (ARDS), and AKI, where precise and individualized fluid management strategies are crucial. Notably, information concerning the utility of the VExUS in the context of sepsis is limited. Determining whether VExUS is associated with clinical outcomes in patients with sepsis could provide a valuable bedside tool to guide therapeutic strategies that optimize fluid management.

The primary objective of this study is to investigate the association between moderate to severe venous congestion, as assessed by VExUS, and the development of AKI in patients with sepsis. The secondary objectives are to assess the association between VExUS and other outcomes (30-day mortality, ICU mortality, and requirement for renal replacement therapy (RRT)), and to describe the prevalence of venous congestion within the first week of sepsis onset.

## Methods

### Patients and study design

This prospective observational study was conducted at ICUs of four tertiary care hospitals in China from April 2023 to August 2024. The study protocol received approval from an independent ethics committee, and written informed consent was obtained from all participants or their legally authorized representatives. The study was conducted in accordance with the 1964 Declaration of Helsinki.

Adult patients were eligible if they were diagnosed with sepsis and stayed in the ICU for at least 24 h after the diagnosis. Sepsis was defined based on Sepsis-3 criteria [10], which requires the evidence of infection and sequential organ failure assessment (SOFA) scores of ≥ 2 from baseline. Septic shock was a subset of sepsis, which was defined as sepsis with persisting hypotension requiring vasopressors to maintain MAP ≥ 65 mmHg and having a serum lactate level > 2 mmol/L despite adequate fluid resuscitation. The exclusion criteria were: (1) age < 18 years; (2) pregnant; (3) poor quality of ultrasound images unsuitable for analysis; (4) end-stage renal disease requiring maintenance dialysis; (5) atrial fibrillation; (6) hepatic cirrhosis; (7) portal vein or hepatic vein thrombosis; (8) history of liver transplant or kidney transplant, and (9) inability to consent without a proxy decision maker.

### Measurements and data collection

Baseline demographic and clinical characteristics were collected at inclusion, including age, sex, acute physiology and chronic health evaluation (APACHE) II score, SOFA score, comorbidities, primary admission diagnosis, primary infectious source, invasive mechanical ventilation use, and vasoactive drug use. The hemodynamic parameters included: heart rate (HR), mean blood pressure (MAP) and CVP. CVP was measured through a central venous catheter indwelling in the internal jugular or subclavian vein. Measurements were taken at the end-expiratory phase with patients in the supine position after zeroing at one-third of the anteroposterior diameter of the chest [11]. The CVP values were averaged over three cardiac cycles. For analysis, each CVP measurement was performed immediately before the VExUS assessment. The biological parameters included: arterial lactate, serum creatinine, blood urea nitrogen (BUN), estimated glomerular filtration rate (eGFR) and total bilirubin. Fluid balance was recorded daily over the first 5 days of ICU admission. Cumulative fluid balance was computed by the addition of daily fluid balances. Echocardiographic, and VExUS examinations were performed at the following time intervals: within 24 h of ICU admission (day 1), 48–72 h following ICU admission (day 3), and 96–120 h following ICU admission (day 5). Clinically relevant outcomes such as 30-day mortality, ICU mortality, ICU length of stay (LOS), AKI during ICU stay, staging of AKI according to Kidney Disease Improving Global Outcomes (KDIGO), and requirement for renal replacement therapy (RRT) were also recorded.

### Ultrasound measurements

Fours fully trained operators (GZC, DTL, LZ and HZF), who were not involved in the management of patients performed ultrasound examinations. Before this study, all operators completed a 4-h online training course and independently performed a minimum of 50 echocardiographic and VExUS examinations to ensure proficiency. All ultrasound images were reviewed off-line by two independent operators (JS and QCL) who experienced in critical care echography and blinded to the study outcomes.

Transthoracic echocardiography was performed using CX-50 (Philips Healthcare, Amsterdam, The Netherlands), Venue Go™ (GE Healthcare, Wuxi City, China) and M9 (Mindray, Shenzhen, China) ultrasound devices with concomitant electrocardiogram measurements. The left ventricular ejection fraction (LVEF) was evaluated visually from apical four-chamber view, and divided into four grades (< 30%; 30–50%; 50–70%; > 70%). The left ventricular outflow tract velocity time integral (VTI) was obtained by placing the pulse-wave Doppler gate at the left ventricular outflow tract in the apical five-chamber view. Right ventricular (RV) function was assessed qualitatively by the ratio between the RV and LV end-diastolic areas, and classified into three grades [12]: normal size (right ventricular end-diastolic area [RVEDA]/left ventricular end-diastolic area [LVEDA] < 0.6), moderately enlarged (RVEDA/LVEDA ≥ 0.6 to < 1), and severely enlarged (RVEDA/LVEDA ≥ 1). The global systolic function of the RV was assessed by measuring the M-mode-derived tricuspid annular plane systolic excursion (TAPSE) from RV-focused apical four-chamber view.

The VExUS scoring system is composed of evaluations of the IVC as well as the pulsed-wave Doppler ultrasound evaluations of the hepatic, portal, and intrarenal venous flow patterns. The analysis of its independent components and calculation of the grading system were conducted as described by Beaubien–Souligny et al. [9]. Their seminal study demonstrated an association between AKI and VExUS (type C) ≥ 2. Accordingly, patients were categorized into two groups: non-congested (VExUS < 2) and congested (VExUS ≥ 2) for further analysis.

### Definitions and outcomes

The primary endpoint was the occurrence of AKI during the ICU stay, as defined by the KDIGO criteria [13]. Baseline serum creatinine was determined by the lowest value recorded within the 12 months before ICU admission; if unavailable, the lowest value measured within the first 24 h of ICU admission was used. To assess the onset of AKI, serum creatinine levels were measured daily, and urinary output was monitored every 6 h during the ICU stay.

The secondary endpoints included 30-day mortality, ICU mortality, and requirement for RRT during the ICU stay. For patients discharged from the ICU within 30 days, telephone follow-up was conducted at 30 days after enrollment to assess clinical outcomes.

### Statistical analyses

Based on a previous study [14], we assumed a prevalence of venous congestion to be 25%. The estimated incidence of AKI in patients with sepsis is 30% [15]. During the study design phase, there were limited prior data quantifying the effect of venous congestion on AKI in sepsis; we set a 30% absolute difference in AKI between patients with venous congestion and without venous congestion to ensure feasibility of enrollment. Therefore, we calculated that a sample of 108 patients would provide a statistical power of 80% and an α-error of 0.05.

Descriptive statistics were calculated to describe the cohort. Quantitative variables are expressed as medians with interquartile ranges (IQR), while categorical variables are presented as frequencies and percentages (%). Patients were stratified into two groups based on VExUS scores (< 2 vs. ≥ 2) and compared across three different time points (days 1, 3, and 5). Quantitative variables were compared using the Mann–Whitney U test, and categorical variables were compared using the chi-square or Fisher’s exact test, as appropriate. To evaluate the association between repeated VExUS measurements (coded as a dichotomous variable: < 2 vs. ≥ 2) and clinical outcomes (AKI, 30-day mortality, ICU mortality, and requirement for RRT), generalized estimating equation (GEE) models were employed. GEE models were developed as an extension of the general linear model to analyze longitudinal and other correlated data. The independent variables incorporated into the model included group, assessment time, and the interaction between group and assessment time. An additional model adjusting for age, sex, chronic kidney disease (CKD) and APACHE II score was constructed to account for baseline risk factors and illness severity. These covariates were chosen a priori to minimize overfitting given the sample size. Linear regression analyses were performed to explore the potential associations between VExUS grade and CVP as well as cumulative fluid balance. Furthermore, to examine the robustness of the estimation, the post hoc sensitivity analyses were conducted using GEE models and logistic regression models to analyze the relationship between VExUS grade (coded as categorical variable: 0, 1, 2, and 3) and all endpoints. A two-tailed *p*-value of ≤ 0.05 was considered statistically significant. All analyses were performed using R version 4.0.2 (R Foundation for Statistical Computing, Vienna, Austria).

## Results

### Baseline characteristics of the study cohort

During the study period, 185 patients were enrolled, of whom 77 (42%) met the exclusion criteria. The primary reasons for exclusion were the inability to obtain analyzable intrarenal venous Doppler waveforms (n = 26) and the presence of atrial fibrillation (n = 22) (Additional file: Supplementary Fig. 1). Of the 108 patients included in the study, a prior creatinine value (within 12 months) was available in 90 patients (83%), whereas for 18 patients (17%) the admission value had to serve as baseline. The demographic characteristics of patients are presented in Table 1. The median age was 74 [61–84] years. The SOFA score at admission was 8 [6–11]. The most frequent ICU admission diagnoses were: respiratory failure (44 patients, 41%), infection of unknown origin (25 patients, 23%) and post-surgical conditions (17 patients, 16%). The primary sources of infection were the respiratory tract (88 patients, 81%) and the intra-abdominal (19 patients, 18%). Eighty-six (80%) patients required mechanical ventilation (MV). Eighty-one (75%) patients presented with septic shock and, 61 patients (56%) received norepinephrine at enrollment.

**Table 1:** Baseline characteristics of the study cohort

Variables	Overall cohort (n = 108)
Age (years), median (IQR)	74 (61–84)
Male, n (%)	77 (71)
APACHE II, median (IQR)	23 (19–28)
SOFA score, median (IQR)	8 (6–11)
Comorbidities, n (%)	
Hypertension	62 (57)
Diabetes	33 (31)
Stroke	21 (19)
Coronary artery disease	17 (16)
Heart failure	14 (13)
Chronic kidney disease	12 (11)
Chronic obstructive pulmonary disease	8 (7)
Main admission diagnosis, n (%)	
Respiratory failure	44 (41)
Infection of unknown origin	25 (23)
Surgical	17 (16)
Neurological	6 (6)
Decompensated heart failure	5 (5)
Trauma	3 (3)
Cardiac arrest	3 (3)
Others	5 (5)
Primary site of infection, n (%)	
Pulmonary	88 (81)
Intra-abdominal	19 (18)
Bloodstream	7 (6)
Urinary	5 (5)
Skin and soft tissue	2 (2)
Central nervous system	1 (1)
Septic shock, n (%)	81 (75)
Invasive MV, n (%)	86 (80)
Vasopressors, n (%)	
Norepinephrine	61 (56)
Epinephrine	6 (6)
Metaraminol	6 (6)
Dopamine	4 (4)
Outcomes	
30-day mortality, n (%)	30 (28)
ICU mortality, n (%)	38 (35)
ICU LOS (days), median (IQR)	12 (7–25)
AKI during ICU stay, n (%)	58 (54)
Staging of AKI according to KDIGO, n (%)	
Stage 1	13 (12)
Stage 2	17 (16)
Stage 3	28 (26)
Requirement for RRT, n (%)	23 (21)

*IQR* interquartile ranges, *APACHE* acute physiology and chronic health evaluation, *SOFA* sequential organ failure assessment, *MV* mechanical ventilation, *ICU* intensive care unit, *LOS* length of stay, *AKI* acute kidney injury, *KDIGO* Kidney Disease Improving Global Outcomes, *RRT* renal replacement therapy

During ICU stay, a total of 58 (54%) patients developed AKI. Of them, 28 (26%) patients were classified as stage 3, 17 (16%) patients as stage 2, and 13 (12%) patients as stage 1. Twenty-three (21%) patients required RRT. Of the 108 patients, 30 (28%) died within 30 days of enrollment, while 38 (35%) died in the ICU. The median ICU LOS was 12 [7–25] days.

### Assessment of VExUS scores

**Fig. 1:**
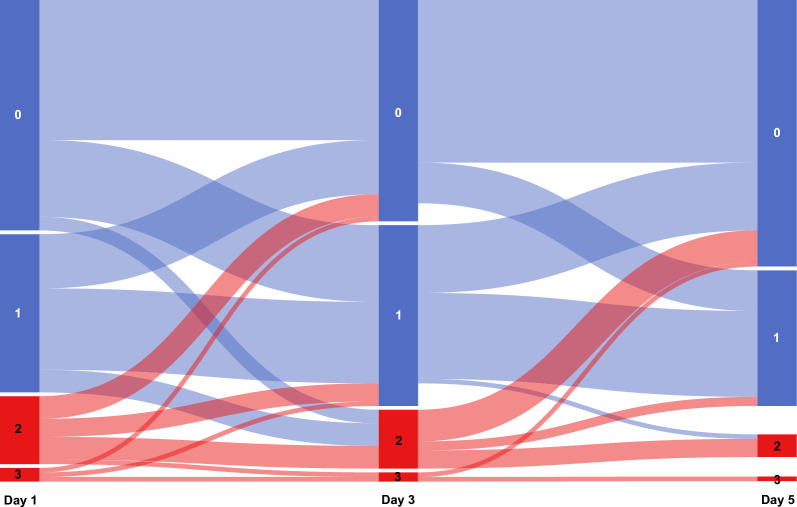
Alluvial diagram showing the temporal trajectory of VExUS scores on days 1, 3, and 5 following ICU admission. The blue color represents patients with VExUS scores < 2. The red color represents patients with VExUS scores ≥ 2

Of the 108 patients analyzed, 18% (19 patients) showed a VExUS score ≥ 2 on day 1 of ICU admission, which progressively decreased to 15% (15 patients) by day 3 and 6% (6 patients) by day 5. Detailed VExUS assessments, and relevant clinical and ultrasound parameters on days 1, 3, and 5 following ICU admission are described in Table 2. The temporal trajectory of VExUS scores on days 1, 3, and 5 following ICU admission is shown in Fig. 1.

**Table 2:** VExUS scores, clinical parameters, and echocardiographic variables at different timepoints

Variables	D1 (n = 108)	D3 (n = 104)	D5 (n = 95)
VExUS grades, n (%)			
0	53 (49)	49 (47)	59 (62)
1	36 (33)	40 (38)	30 (32)
2	16 (15)	13 (13)	5 (5)
3	3 (3)	2 (2)	1 (1)
Hepatic vein pattern, n (%)			
Normal	86 (80)	83 (80)	78 (82)
Mildly abnormal	12 (11)	16 (15)	12 (13)
Severely abnormal	10 (9)	5 (5)	5 (5)
Portal vein pattern, n (%)			
Normal	60 (56)	43 (41)	48 (51)
Mildly abnormal	35 (32)	54 (52)	43 (45)
Severely abnormal	13 (12)	7 (7)	4 (4)
Intrarenal venous pattern, n (%)			
Normal	55 (51)	49 (47)	52 (55)
Mildly abnormal	50 (46)	49 (47)	39 (41)
Severely abnormal	3 (3)	6 (6)	4 (4)
HR (bpm), median (IQR)	96 (77–112)	90 (78–109)	95 (81–108)
MAP (mmHg), median (IQR)	79 (69–92)	82 (75–94)	83 (75–94)
Creatinine (μmol/L), median (IQR)	91.5 (61.5–140.3)	83 (59.8–141.5)	81 (60–146.5)
Cumulative fluid balance (mL), median (IQR)	692 (−79–1618)	1529 (−226–3057)	2666 (476–4189)
Maximum IVC diameter (cm), median (IQR)	2 (1.6–2.1)	2 (1.7–2.2)	1.8 (1.6–2.1)
IVC diameter ≥ 2 cm, n (%)	55 (51)	55 (53)	36 (38)
PI (%), median (IQR)	28 (20–39)	32 (21–40)	30 (21–38)
LVOT-VTI (cm), median (IQR)	21.8 (16.1–24.9)	21.8 (17.4–25.7)	21.9 (17.8–25.7)
TAPSE (cm), median (IQR)	1.9 (1.7–2.2)	1.9 (1.7–2.3)	1.9 (1.7–2.3)
RVEDA/LVEDA ≥ 0.6, n (%)	11 (10)	12 (12)	11 (12)
Visual LVEF, n (%)			
< 30%	1 (1)	2 (2)	2 (2)
30–50%	18 (17)	19 (18)	18 (19)
50–70%	79 (73)	76 (73)	67 (71)
> 70%	10 (9)	7 (7)	8 (8)

*VExUS* Venous Excess Ultrasound, *HR* heart rate, *bmp* beat per minute, *IQR* interquartile ranges, *MAP* mean arterial pressure, *IVC* inferior vena cava, *PI* pulsatility index, *LVOT* left ventricular outflow tract, *VTI* velocity time integral, *TAPSE* tricuspid annular plane systolic excursion, *RVEDA* right ventricular end-diastolic area, *LVEDA* left ventricular end-diastolic area, *LVEF* left ventricular ejection fraction

Clinical and ultrasound parameters for patients with VExUS < 2 and VExUS ≥ 2 on days 1, 3, and 5 following ICU admission are presented in the Additional file: Supplementary Table 1.

**Fig. 2:**
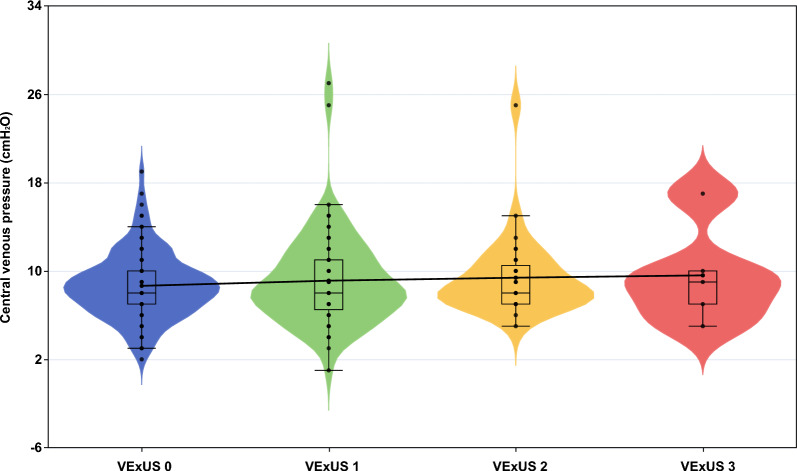
Violin plot depicting the distribution of CVP across different VExUS grades

Due to the absence of central venous catheters in the subclavian or jugular veins, 75 CVP measurements were unavailable, with 232 CVP measurements available for the final analysis. There were no significant differences in CVP between patients with different VExUS scores (*p* = 0.922) (Fig. 2), and no significant correlation was found between CVP and VExUS scores (coefficient: − 0.019, 95% confidence interval (CI) −0.01 to 0.05, *p* = 0.204). Similarly, we did not observe a significant relationship between cumulative fluid balance and VExUS (coefficient: 9.59, 95% CI −390.65 to 409.83, *p* = 0.963).

### Association between VExUS scores and clinical outcomes

Table 3 showed the association between VExUS ≥ 2 and clinical outcomes. After adjusting for age, sex, CKD and APACHE II score, the GEE model did not show statistically significant association between VExUS ≥ 2 and AKI (adjusted odds ratio (OR) 1.82, 95% CI 0.62–5.31, *p* = 0.274). The secondary endpoint analyses gave similar results to those obtained in the primary endpoint analysis (Table 3). In post-hoc sensitivity analyses, we applied GEE models and logistic regression models respectively, confirming the lack of significant association between VExUS and clinical outcomes (Additional file: Supplementary Table 2 and Supplementary Table 3). Results of the septic shock subgroup analyses were similar to those from the analysis of the overall cohort (Additional file: Supplementary Table 4).

**Table 3:** Association between VExUS ≥ 2 and clinical outcomes

Outcomes	Generalized estimating equation model					
	Unadjusted OR (95% CI)			Adjusted* OR (95% CI)		
	OR	95% CI	*p*-value	OR	95% CI	*p*-value
AKI	2.12	0.74–6.07	0.162	1.82	0.62–5.31	0.274
30-day mortality	0.91	0.3–2.81	0.875	0.82	0.28–2.4	0.711
ICU mortality	1.43	0.52–3.93	0.488	1.12	0.41–3.04	0.82
Requirement for RRT	2.66	0.91–7.82	0.075	2.29	0.68–7.64	0.179

*OR* odds ratio, *CI* confidence interval, *AKI* acute kidney injury, *ICU* intensive care unit, *RRT* renal replacement therapy

^*^adjusted for age, sex, CKD and APACHE II score. The reference level was VExUS < 2

## Discussion

The key findings of the present study can be summarized as follows: (1) Approximately 20% of sepsis patients who underwent VExUS assessments within 24 h of ICU admission had an elevated VExUS score (grade ≥ 2) indicating significant venous congestion, with the prevalence progressively decreasing over the first 5 days. (2) No significant correlation was found between VExUS scores and CVP. (3) VExUS scores was not associated with AKI or mortality (30-day and ICU mortality) in critically ill patients with sepsis.

Our study showed that venous congestion assessed by VExUS was present in less than 20% of sepsis patients within the first 24 h of ICU admission. Specifically, moderate and severe venous congestion was observed in 15% and 3% of patients, respectively. This finding is consistent with existing research involving non-cardiac patients. Andrei et al. reported a 22% prevalence of abnormal VExUS scores in a cohort of general critically ill patients at ICU admission [14]. Similarly, in another study involving 75 patients with septic shock, Prager et al. found that the incidence of severe venous congestion (VExUS ≥ 2) was 19% [16]. Indeed, VExUS scores were not associated with cumulative fluid balance in our study. Although this result may seem unexpected, it is consistent with published studies that indicate there is no clear link between VExUS and net fluid balance [16, 17]. VExUS do not simply reflect the volume of fluid administered; rather, it is influenced by several factors in the sepsis patients, such as systemic inflammation, cardiac function, vasodilation, and capillary leak syndrome, which may contribute to the relative depletion of intravascular volume [18, 19]. Additionally, sepsis patients may exhibit different cardiovascular phenotypes [20]. VExUS only identifies the intravascular venous congestion (elevated CVP), but it does not directly capture interstitial congestion (tissue edema), which is common in sepsis. For example, a patient may have a normal VExUS score (no signs of high venous pressure) yet still suffer severe peripheral edema and organ dysfunction from capillary leak. This discrepancy underscores the challenges in evaluating fluid tolerance in sepsis, as venous congestion is affected by various factors. Interestingly, recent studies have shown VExUS correlating well with invasive right atrial pressures (RAP) measurements. Longino et al. assessed associations between VExUS grade and RAP, as measured by right heart catheterization (RHC), in a cohort of 51 patients. They found a significant positive association between RAP and VExUS grade (*p* < 0.001, R^2^ = 0.68) [21]. Additionally, their follow-up study among 81 patients showed similar correlations between VExUS grade and RAP [22]. However, in our sepsis cohort, we observed no correlation between VExUS grade and CVP. In the ICU, CVP measurements are affected by several factors, including thoracic, pericardial, and abdominal pressures, as well as ventilator settings, complicating their interpretation. Furthermore, in our study, the ultrasound parameters assessing left- and right-sided cardiac function (such as LVEF, TAPSE, and the RVEDA/LVEDA ratio) were within normal ranges for the majority of patients, which may partly explain the absence of correlation between VExUS and CVP. Previous research has shown that a higher VExUS grade (VExUS ≥ 2) is primarily associated with cardiac comorbidities and cardiac function [23]. Therefore, challenges remain in evaluating and interpreting venous congestion in complex clinical contexts. Although VExUS is a valuable tool, it should be used alongside clinical and other hemodynamic assessments to optimize fluid management strategies for patients with sepsis.

We observed no significant association between VExUS and AKI, which is in line with previous studies in septic shock or mixed ICU patients [14, 16]. Venous congestion is a well-established risk factor for AKI in patients with cardiac dysfunction [24]. In these patients, elevated venous pressures lead to impaired renal perfusion, triggering kidney damage via mechanisms such as a reduced GFR and increased tubular cell injury due to stasis of venous blood [25–27]. However, the pathophysiology of sepsis-related AKI is considerably more complex. It involves a complex interplay of multifactorial pathophysiological mechanisms, including hemodynamic alterations, ischemia–reperfusion injury, inflammatory responses, endothelial dysfunction, and microcirculatory dysfunction [15, 28]. These mechanisms are likely the primary drivers of sepsis-related AKI [29]. It is also important to acknowledge that the relationship between venous congestion and AKI may be bidirectional. Elevated venous pressures can worsen kidney function, but conversely, early AKI (from other sepsis-related mechanisms) could lead to fluid retention and venous congestion. In our observational study, association does not prove causation. Therefore, VExUS has limited utility in predicting AKI in sepsis or mixed ICU settings, as opposed to its established value in cardiac patients.

Our study explores the association between VExUS and clinical outcomes in patients with sepsis, while also providing important insights for its broader application in different patient populations. Given the harmful effects of fluid overload, clinicians are eager to find a tool, preferably a non-invasive, that can objectively quantifying fluid overload and guiding the optimization of volume management. VExUS represents a promising advancement in the non-invasive assessment of right-sided venous flow. However, the interpretation of VExUS findings in the complex ICU setting may be challenging due to the heterogeneous pathophysiological mechanisms underlying venous congestion across different patient subgroups, such as those with cirrhosis, significant tricuspid regurgitation, chronic kidney disease, or those receiving positive pressure ventilation. The findings of the present study may prompt us to reconsider the clinical utility of VExUS in specific ICU populations, while also encourage more high-quality studies to explore the pathophysiological mechanism of venous congestion in different patient populations.

This study has several limitations. First, the high selectivity of the study population limits the generalizability of the findings. Second, we did not evaluate the intra- and inter-operator variability for the ultrasound examinations. However, all operators were trained in a standardized VExUS protocol and all ultrasound examinations were confirmed offline by two independent operators, which may reduce the intra- and inter-operator variability to some extent. Third, although all patients underwent an initial VExUS assessment within 24 h of ICU admission, the timing of the assessments varied (e.g., some were assessed early during fluid resuscitation, while others were assessed later). This variability may underestimate the incidence of early venous congestion in patients with sepsis. However, serial VExUS assessments were conducted at multiple time points to minimize the limitations of single time point assessments. Fourth, 30 patients were excluded due to incomplete VExUS assessments, primarily because analyzable intrarenal venous flow Doppler waveforms could not be obtained. However, this is consistent with the real-world clinical scenarios, as Spiegel et al. reported that 25.4% of general ICU patients were unable to obtain analyzable intrarenal venous Doppler signals [30]. Finally, the low proportion of patients with venous congestion (only 18% had VExUS ≥ 2) likely reduced the statistical power of the study to detect meaningful associations. Due to the limited prior data quantifying the effect of venous congestion on AKI in sepsis patients, our sample size was calculated based on an expected 25% congestion prevalence, which we overestimated. As a result, our negative findings could be due in part to the small number of congested patients. Given the limitations in sample size, larger-scale multicenter studies are needed to further clarify the role of VExUS in predicting clinical outcomes in patients with sepsis.

## Conclusion

In critically ill patients with sepsis, approximately 20% exhibit venous congestion assessed by VExUS within the first 24 h of ICU admission, with the proportion progressively decreasing over the subsequent 5 days. Moderate to severe venous congestion is not associated with AKI or mortality (30-day and ICU mortality). Further studies with larger and more diverse ICU populations are needed to validate the utility of VExUS in assessing venous congestion.

## Supplementary Information

Supplementary material 1.

## Data Availability

The datasets used and/or analyzed during the current study are available from the corresponding author on reasonable request.

## Abbreviations

AKI: Acute kidney injury
ARDS: Acute respiratory distress syndrome
APACHE: Acute physiology and chronic health evaluation
BUN: Blood urea nitrogen
CKD: Chronic kidney disease
CVP: Central venous pressure
CI: Confidence interval
eGFR: Estimated glomerular filtration rate
GEE: Generalized estimating equation
HR: Heart rate
ICU: Intensive care unit
IQR: Interquartile ranges
IVC: Inferior vena cava
KDIGO: Kidney Disease Improving Global Outcomes
LOS: Length of stay
LVEF: Left ventricular ejection fraction
LVEDA: Left ventricular end-diastolic area
LVOT: Left ventricular outflow tract
MAP: Mean blood pressure
MV: Mechanical ventilation
OR: Odds ratio
PI: Pulsatility index
RHC: Right heart catheterization
RRT: Renal replacement therapy
RV: Right ventricular
RVEDA: Right ventricular end-diastolic area
SOFA: Sequential organ failure assessment
SSC: Surviving Sepsis Campaign
TAPSE: Tricuspid annular plane systolic excursion
VExUS: Venous Excess Ultrasound
VTI: Velocity time integral

## References

[1] Meyer NJ, Prescott HC. Sepsis and septic shock. N Engl J Med. 2024;391:2133–46. 39774315 10.1056/NEJMra2403213

[2] Malbrain MLNG, Van Regenmortel N, Saugel B, De Tavernier B, Van Gaal P-J, Joannes-Boyau O, et al. Principles of fluid management and stewardship in septic shock: it is time to consider the four D’s and the four phases of fluid therapy. Ann Intensive Care. 2018;8:66. 29789983 10.1186/s13613-018-0402-x PMC5964054

[3] Zampieri FG, Bagshaw SM, Semler MW. Fluid therapy for critically ill adults with sepsis: a review. JAMA. 2023;329:1967. 37314271 10.1001/jama.2023.7560

[4] Sakr Y, Rubatto Birri PN, Kotfis K, Nanchal R, Shah B, Kluge S, et al. Higher fluid balance increases the risk of death from sepsis: results from a large international audit. Crit Care Med. 2017;45:386–94. 27922878 10.1097/CCM.0000000000002189

[5] Andrews B, Semler MW, Muchemwa L, Kelly P, Lakhi S, Heimburger DC, et al. Effect of an early resuscitation protocol on in-hospital mortality among adults with sepsis and hypotension: a randomized clinical trial. JAMA. 2017;318:1233–40. 28973227 10.1001/jama.2017.10913 PMC5710318

[6] Tackaert T, Van Moorter N, De Mey N, Demeyer I, De Decker K. The association between increasing fluid balance, acute kidney injury and mortality in patients with sepsis and septic shock: a retrospective single center audit. J Crit Care. 2023;78:154367. 37494863 10.1016/j.jcrc.2023.154367

[7] Chen C-Y, Zhou Y, Wang P, Qi E-Y, Gu W-J. Elevated central venous pressure is associated with increased mortality and acute kidney injury in critically ill patients: a meta-analysis. Crit Care. 2020;24: 80. 32138764 10.1186/s13054-020-2770-5 PMC7059303

[8] Kattan E, Castro R, Miralles-Aguiar F, Hernández G, Rola P. The emerging concept of fluid tolerance: a position paper. J Crit Care. 2022;71: 154070. 35660844 10.1016/j.jcrc.2022.154070

[9] Beaubien-Souligny W, Rola P, Haycock K, Bouchard J, Lamarche Y, Spiegel R, et al. Quantifying systemic congestion with point-of-care ultrasound: development of the venous excess ultrasound grading system. Ultrasound J. 2020;12: 16. 32270297 10.1186/s13089-020-00163-w PMC7142196

[10] Singer M, Deutschman CS, Seymour CW, Shankar-Hari M, Annane D, Bauer M, et al. The third international consensus definitions for sepsis and septic shock (sepsis-3). JAMA. 2016;315:801. 26903338 10.1001/jama.2016.0287 PMC4968574

[11] Lloyd-Donald P, Fujino M, Waldman B, Miles LF. Measurement and interpretation of central venous pressure: a narrative review. Anaesthesia. 2025. 10.1111/anae.16633. 40457939 10.1111/anae.16633 PMC12351226

[12] Rudski LG, Lai WW, Afilalo J, Hua L, Handschumacher MD, Chandrasekaran K, et al. Guidelines for the echocardiographic assessment of the right heart in adults: a report from the American society of echocardiography endorsed by the European association of echocardiography, a registered branch of the European society of cardiology, and the Canadian society of echocardiography. J Am Soc Echocardiogr. 2010;23:685–713. 20620859 10.1016/j.echo.2010.05.010

[13] Kellum JA, Lameire N, for the KDIGO AKI Guideline Work Group. Diagnosis, evaluation, and management of acute kidney injury: a KDIGO summary (part 1). Crit Care. 2013;17:204. 23394211 10.1186/cc11454 PMC4057151

[14] Andrei S, Bahr P-A, Nguyen M, Bouhemad B, Guinot P-G. Prevalence of systemic venous congestion assessed by venous excess ultrasound grading system (VExUS) and association with acute kidney injury in a general ICU cohort: a prospective multicentric study. Crit Care. 2023;27: 224. 37291662 10.1186/s13054-023-04524-4 PMC10249288

[15] Peerapornratana S, Manrique-Caballero CL, Gómez H, Kellum JA. Acute kidney injury from sepsis: Current concepts, epidemiology, pathophysiology, prevention and treatment. Kidney Int. 2019;96:1083–99. 31443997 10.1016/j.kint.2019.05.026 PMC6920048

[16] Prager R, Arntfield R, Wong MYS, Ball I, Lewis K, Rochwerg B, et al. Venous congestion in septic shock quantified with point-of-care ultrasound: a pilot prospective multicentre cohort study. Can J Anaesth. 2024;71:640–9. 38548949 10.1007/s12630-024-02717-1

[17] Muñoz F, Born P, Bruna M, Ulloa R, González C, Philp V, et al. Coexistence of a fluid responsive state and venous congestion signals in critically ill patients: a multicenter observational proof-of-concept study. Crit Care. 2024;28:52. 38374167 10.1186/s13054-024-04834-1 PMC10877871

[18] Saravi B, Goebel U, Hassenzahl LO, Jung C, David S, Feldheiser A, et al. Capillary leak and endothelial permeability in critically ill patients: a current overview. Intensive Care Med Exp. 2023;11:96. 38117435 10.1186/s40635-023-00582-8 PMC10733291

[19] Iba T, Maier CL, Helms J, Ferrer R, Thachil J, Levy JH. Managing sepsis and septic shock in an endothelial glycocalyx-friendly way: from the viewpoint of surviving sepsis campaign guidelines. Ann Intensive Care. 2024;14:64. 38658435 10.1186/s13613-024-01301-6 PMC11043313

[20] Messina A, Vieillard-Baron A. How we could use critical care echocardiography in the assessment of and management of cardiovascular phenotypes in septic shock: the good, the bad, and the ugly profiles. Intensive Care Med. 2025;51:397–400. 39833496 10.1007/s00134-025-07782-8

[21] Longino A, Martin K, Leyba K, Siegel G, Gill E, Douglas IS, et al. Correlation between the VExUS score and right atrial pressure: a pilot prospective observational study. Crit Care. 2023;27:205. 37237315 10.1186/s13054-023-04471-0 PMC10223840

[22] Longino A, Martin K, Leyba K, Siegel G, Thai TN, Riscinti M, et al. Prospective evaluation of venous excess ultrasound for estimation of venous congestion. Chest. 2024;165:590–600. 37813180 10.1016/j.chest.2023.09.029 PMC11317813

[23] Andrei S, Nguyen M, Bouhemad B, Guinot P-G. High VExUS grades are linked to cardiac function in general ICU patients. Eur Heart J Acute Cardiovasc Care. 2025;14:24–30. 10.1093/ehjacc/zuae126 39520388

[24] Lopez MG, Shotwell MS, Morse J, Liang Y, Wanderer JP, Absi TS, et al. Intraoperative venous congestion and acute kidney injury in cardiac surgery: an observational cohort study. Br J Anaesth. 2021;126:599–607. 33549321 10.1016/j.bja.2020.12.028 PMC8014941

[25] Yu Y, Li C, Zhu S, Jin L, Hu Y, Ling X, et al. Diagnosis, pathophysiology and preventive strategies for cardiac surgery-associated acute kidney injury: a narrative review. Eur J Med Res. 2023;28:45. 36694233 10.1186/s40001-023-00990-2 PMC9872411

[26] Rangaswami J, Bhalla V, Blair JEA, Chang TI, Costa S, Lentine KL, et al. Cardiorenal syndrome: classification, pathophysiology, diagnosis, and treatment strategies: a scientific statement from the American Heart Association. Circulation. 2019;139: e840–78. 30852913 10.1161/CIR.0000000000000664

[27] Islas-Rodríguez JP, Miranda-Aquino T, Romero-González G, Hernández-Del Rio J, Camacho-Guerrero JR, Covarrubias-Villa S, et al. Effect on kidney function recovery guiding decongestion with VExUS in patients with cardiorenal syndrome 1: a randomized control trial. Cardiorenal Med. 2024;14:1–11. 38061346 10.1159/000535641

[28] Bellomo R, Kellum JA, Ronco C, Wald R, Martensson J, Maiden M, et al. Acute kidney injury in sepsis. Intensive Care Med. 2017;43:816–28. 28364303 10.1007/s00134-017-4755-7

[29] Post EH, Kellum JA, Bellomo R, Vincent J-L. Renal perfusion in sepsis: from macro- to microcirculation. Kidney Int. 2017;91:45–60. 27692561 10.1016/j.kint.2016.07.032

[30] Spiegel R, Teeter W, Sullivan S, Tupchong K, Mohammed N, Sutherland M, et al. The use of venous doppler to predict adverse kidney events in a general ICU cohort. Crit Care. 2020;24: 615. 33076961 10.1186/s13054-020-03330-6 PMC7574322

