# Supplementary material for: Association between the Venous Excess Ultrasound (VExUS) score and acute kidney injury in critically ill patients with sepsis: a multicenter prospective observational study
Source: Ann Intensive Care. 2025 Jul 23;15:105. doi: 10.1186/s13613-025-01529-w (PMC12287484; doi:10.1186/s13613-025-01529-w)
Supplement: Supplementary file 1 — Supplementary material 1. [file 13613_2025_1529_MOESM1_ESM.docx]

**Additional File**

**Association between the Venous Excess Ultrasound (VExUS) score and acute kidney injury in critically ill patients with sepsis: a multicenter prospective observational study**

**Supplementary Table 1A** Clinical characteristics of patients with VExUS < 2 and VExUS ≥ 2 on day 1 following ICU admission

| **Variables** | **All patients**  **(n = 108)** | **VExUS < 2**  **(n = 89)** | **VExUS** **≥ 2**  **(n = 19)** | ***p*-value** |
| --- | --- | --- | --- | --- |
| **Clinical variables** |  |  |  |  |
| HR (bpm), median (IQR) | 96 (77–113) | 95 (75–108) | 110 (78–122) | 0.276 |
| MAP (mmHg), median (IQR) | 79 (69–92) | 79 (69–92) | 81 (69–87) | 0.965 |
| CVP (cmH_2_O), median (IQR)* | 8 (7–11) | 8 (7–11) | 9 (7–11) | 0.485 |
| Lactate (mmol/L), median (IQR) | 2 (1.3–3.2) | 2 (1.3–3.2) | 2.4 (1.4–3.9) | 0.372 |
| pO_2_/FiO_2_, median (IQR) | 250 (177–324) | 252 (177–326) | 243 (179–290) | 0.41 |
| Creatinine (μmol/L), median (IQR) | 91.5 (61.3–140.5) | 86 (61–136) | 132 (84–184) | 0.053 |
| BUN (mmol/L), median (IQR) | 8.9 (6.2–14.8) | 8.5 (6.2–13.5) | 13.9 (5.2–23) | 0.149 |
| eGFR (mL/min/1.73 m^2^), median (IQR) | 63.8 (37.8–93.3) | 69 (41.5–94.8) | 41.4 (30.4–70.8) | 0.023 |
| Bilirubin (μmol/L), median (IQR) | 14.1 (9.7–20.3) | 14.5 (10.3–20.1) | 11.9 (9.2–22.5) | 0.278 |
| 24 h fluid balance (mL), median (IQR) | 692 (-79–1618) | 580 (-102–1404) | 1,164 (470–2052) | 0.133 |
| **Ultrasound variables** |  |  |  |  |
| Maximum IVC diameter (cm), median (IQR) | 2 (1.6–2.1) | 1.8 (1.6–2.1) | 2.1 (2–2.2) | 0.001 |
| PI (%), median (IQR) | 0.28 (0.2–0.39) | 0.26 (0.19–0.35) | 0.55 (0.31–0.59) | <0.001 |
| TAPSE (cm), median (IQR) | 1.9 (1.7–2.2) | 1.9 (1.7–2.2) | 1.8 (1.5–2) | 0.198 |
| LVOT-VTI (cm), median (IQR) | 22 (16–25) | 21 (16–25) | 24 (16–28) | 0.537 |
| Visual LVEF, n (%) |  |  |  | 0.204 |
| < 30% | 1 (1) | 1 (1) | 0 (0) |  |
| 30% – 50% | 18 (17) | 12 (14) | 6 (32) |  |
| 50% – 70% | 79 (73) | 68 (76) | 11 (58) |  |
| > 70% | 10 (9) | 8 (9) | 2 (11) |  |
| **Clinical outcomes** |  |  |  |  |
| 30-day mortality, n (%) | 30 (28) | 25 (28) | 5 (26) | 0.875 |
| AKI within ICU stay, n (%) | 58 (54) | 45 (51) | 13 (68) | 0.156 |
| ICU mortality, n (%) | 38 (35) | 30 (34) | 8 (42) | 0.487 |
| ICU length of stay (days), median (IQR) | 12 (7–25) | 12 (6–23) | 10 (8–33) | 0.569 |
| MV duration (days), median (IQR) | 8 (3–19) | 8 (2–18) | 8 (7–21) | 0.552 |
| Staging of AKI according to KDIGO, n (%) |  |  |  | 0.395 |
| Stage 1 | 13 (12) | 12 (13) | 1 (5) |  |
| Stage 2 | 17 (16) | 12 (13) | 5 (26) |  |
| Stage 3 | 28 (26) | 21 (124) | 7 (37) |  |
| RRT requirement within ICU stay, n (%) | 23 (21) | 16 (18) | 7 (37) | 0.118 |

*VExUS* Venous Excess Ultrasound; *HR* heart rate; *bmp* beat per minute; *IQR* interquartile range; *MAP* mean arterial pressure; *CVP* central venous pressure; *pO_2_* partial pressure of arterial oxygen; *FiO_2_* fraction of inspiratory oxygen; *BUN* blood urea nitrogen; *eGFR* estimated glomerular filtration rate; *IVC* inferior vena cava; *PI* pulsatility index; *TAPSE* tricuspid annular plane systolic excursion; *LVOT* left ventricular outflow tract; *VTI* velocity time integral; *LVEF* left ventricular ejection fraction; *AKI* acute kidney injury; *ICU* intensive care unit; *MV* mechanical ventilation; *KDIGO* Kidney Disease Improving Global Outcomes; *RRT* renal replacement therapy

* CVP missing for 24 patients (23 were in the VExUS < 2 group and 1 was in the VExUS **≥** 2 group)

**Supplementary Table 1B** Clinical characteristics of patients with VExUS < 2 and VExUS ≥ 2 on day 3 following ICU admission

| **Variables** | **All patients**  **(n = 104)** | **VExUS < 2**  **(n = 89)** | **VExUS ≥ 2**  **(n = 15)** | ***p*-value** |
| --- | --- | --- | --- | --- |
| **Clinical variables** |  |  |  |  |
| HR (bpm), median (IQR) | 90 (78–110) | 92 (82–110) | 78 (70–108) | 0.27 |
| MAP (mmHg), median (IQR) | 82 (75–94) | 84 (74–94) | 80 (75–89) | 0.585 |
| CVP (cmH_2_O), median (IQR)* | 9 (7–10) | 9 (7–10) | 9 (8–10) | 0.893 |
| Lactate (mmol/L), median (IQR) | 1.5 (1.1–2) | 1.4 (1–1.9) | 1.7 (1.2–2.6) | 0.279 |
| pO_2_/FiO_2_, median (IQR) | 283 (222–367) | 282 (220–363) | 285 (239–386) | 0.588 |
| Creatinine (μmol/L), median (IQR) | 83 (59.5–142) | 77 (59–133) | 134 (71–198) | 0.022 |
| BUN (mmol/L), median (IQR) | 10.2 (6.5–16.4) | 9.1 (6.5–15.3) | 14.5 (7.4–20.6) | 0.055 |
| eGFR (mL/min/1.73 m^2^), median (IQR) | 77.4 (37.9–97.2) | 82.9 (44.6–98) | 45.1 (24.5–76.5) | 0.004 |
| Bilirubin (μmol/L), median (IQR) | 13.7 (8.7–20.6) | 13.8 (8.7–22.4) | 12.5 (8.6–16.4) | 0.592 |
| 72 h fluid balance (mL), median (IQR) | 1529 (-226–3057) | 1550 (-194–3025) | 944 (57–3024) | 0.89 |
| **Ultrasound variables** |  |  |  |  |
| Maximum IVC diameter (cm), median (IQR) | 2 (1.7–2.2) | 1.9 (1.7–2.2) | 2.1 (2.1–2.2) | 0.006 |
| PI (%), median (IQR) | 0.32 (0.21–0.4) | 0.31 (0.2–0.38) | 0.43 (0.33–0.53) | 0.001 |
| TAPSE (cm), median (IQR) | 1.9 (1.7–2.3) | 2 (1.7–2.4) | 1.8 (1.6–1.9) | 0.057 |
| LVOT-VTI (cm), median (IQR) | 22 (17–26) | 22 (17–26) | 21 (18–23) | 0.634 |
| Visual LVEF, n (%) |  |  |  | 0.338 |
| < 30% | 2 (2) | 1 (1) | 1 (7) |  |
| 30% – 50% | 19 (18) | 16 (18) | 3 (20) |  |
| 50% – 70% | 76 (73) | 65 (73) | 11 (73) |  |
| > 70% | 7 (7) | 7 (8) | 0 (0) |  |
| **Clinical outcomes** |  |  |  |  |
| 30-day mortality, n (%) | 28 (27) | 22 (25) | 6 (40) | 0.224 |
| AKI within ICU stay, n (%) | 55 (53) | 44 (49) | 11 (73) | 0.086 |
| ICU mortality, n (%) | 36 (35) | 28 (32) | 8 (53) | 0.1 |
| ICU length of stay (days), median (IQR) | 12 (7–27) | 12 (7–24) | 13 (8–36) | 0.384 |
| MV duration (days), median (IQR) | 8 (4–21) | 8 (3–18) | 13 (7–33) | 0.049 |
| Staging of AKI according to KDIGO, n (%) |  |  |  | 0.195 |
| Stage 1 | 13 (13) | 9 (10) | 4 (27) |  |
| Stage 2 | 17 (16) | 16 (18) | 1 (7) |  |
| Stage 3 | 25 (24) | 19 (21) | 6 (40) |  |
| RRT requirement within ICU stay, n (%) | 20 (19) | 16 (18) | 4 (27) | 0.481 |

*VExUS* Venous Excess Ultrasound; *HR* heart rate; *bmp* beat per minute; *IQR* interquartile range; *MAP* mean arterial pressure; *CVP* central venous pressure; *pO_2_* partial pressure of arterial oxygen; *FiO_2_* fraction of inspiratory oxygen; *BUN* blood urea nitrogen; *eGFR* estimated glomerular filtration rate; *IVC* inferior vena cava; *PI* pulsatility index; *TAPSE* tricuspid annular plane systolic excursion; *LVOT* left ventricular outflow tract; *VTI* velocity time integral; *LVEF* left ventricular ejection fraction; *AKI* acute kidney injury; *ICU* intensive care unit; *MV* mechanical ventilation; *KDIGO* Kidney Disease Improving Global Outcomes; *RRT* renal replacement therapy

* CVP missing for 27 patients (24 were in the VExUS < 2 group and 3 were in the VExUS **≥** 2 group)

**Supplementary Table 1C** Clinical characteristics of patients with VExUS < 2 and VExUS ≥ 2 on day 5 following ICU admission

| **Variables** | **All patients**  **(n = 95)** | **VExUS < 2**  **(n = 89)** | **VExUS ≥ 2**  **(n = 6)** | ***p*-value** |
| --- | --- | --- | --- | --- |
| **Clinical variables** |  |  |  |  |
| HR (bpm), median (IQR) | 95 (80–108) | 95 (82–108) | 79 (70–113) | 0.396 |
| MAP (mmHg), median (IQR) | 83 (75–94) | 82 (75–93) | 92 (87–96) | 0.183 |
| CVP (cmH_2_O), median (IQR)* | 8 (6–10) | 8 (6–10) | 8 (5–9) | 0.581 |
| Lactate (mmol/L), median (IQR) | 1.5 (1–2.2) | 1.5 (1–2.2) | 1.4 (1–1.6) | 0.343 |
| pO_2_/FiO_2_, median (IQR) | 280 (208–364) | 280 (208–363) | 272 (214–382) | 0.708 |
| Creatinine (μmol/L), median (IQR) | 81 (60– 147) | 79 (60– 132) | 171.5 (65–179) | 0.124 |
| BUN (mmol/L), median (IQR) | 11.4 (7.6–19.6) | 11.3 (7.6–18.7) | 19.4 (7.6–25.5) | 0.409 |
| eGFR (mL/min/1.73 m^2^), median (IQR) | 70.5 (35.7–101.7) | 70.9 (38.1–101.7) | 31.2 (25.5–78.8) | 0.191 |
| Bilirubin (μmol/L), median (IQR) | 14.6 (9.7–24.5) | 15.1 (9.8–24.7) | 10.9 (9–14) | 0.132 |
| 120 h fluid balance (mL), median (IQR) | 2666 (476–4189) | 2827 (457–4220) | 1446 (703–1836) | 0.44 |
| **Ultrasound variables** |  |  |  |  |
| Maximum IVC diameter (cm), median (IQR) | 1.8 (1.6–2.1) | 1.8 (1.6–2.1) | 2.3 (2.1–2.5) | 0.003 |
| PI (%), median (IQR) | 0.3 (0.21–0.37) | 0.29 (0.21–0.37) | 0.35 (0.32–0.37) | 0.176 |
| TAPSE (cm), median (IQR) | 1.9 (1.7–2.3) | 1.9 (1.7–2.2) | 2 (1.9–2.4) | 0.534 |
| LVOT-VTI (cm), median (IQR) | 22 (18–26) | 21 (18–24) | 26 (23–31) | 0.153 |
| Visual LVEF, n (%) |  |  |  | 0.033 |
| < 30% | 2 (2) | 1 (1) | 1 (17) |  |
| 30% – 50% | 18 (19) | 16 (18) | 2 (33) |  |
| 50% – 70% | 67 (71) | 65 (73) | 2 (33) |  |
| > 70% | 8 (8) | 7 (8) | 1 (17) |  |
| **Clinical outcomes** |  |  |  |  |
| 30-day mortality, n (%) | 27 (28) | 25 (28) | 2 (33) | >0.999 |
| AKI within ICU stay, n (%) | 54 (57) | 49 (55) | 5 (83) | 0.231 |
| ICU mortality, n (%) | 35 (37) | 33 (37) | 2 (33) | >0.999 |
| ICU length of stay (days), median (IQR) | 13 (9–30) | 13 (9–28) | 17 (8–32) | 0.976 |
| MV duration (days), median (IQR) | 10 (5–23) | 10 (4–22) | 17 (7–28) | 0.386 |
| Staging of AKI according to KDIGO, n (%) |  |  |  | 0.846 |
| Stage 1 | 13 (14) | 12 (13) | 1 (17) |  |
| Stage 2 | 17 (18) | 16 (18) | 1 (17) |  |
| Stage 3 | 24 (25) | 21 (24) | 3 (50) |  |
| RRT requirement within ICU stay, n (%) | 20 (21) | 18 (20) | 2 (33) | 0.603 |

*VExUS* Venous Excess Ultrasound; *HR* heart rate; *bmp* beat per minute; *IQR* interquartile range; *MAP* mean arterial pressure; *CVP* central venous pressure; *pO_2_* partial pressure of arterial oxygen; *FiO_2_* fraction of inspiratory oxygen; *BUN* blood urea nitrogen; *eGFR* estimated glomerular filtration rate; *IVC* inferior vena cava; *PI* pulsatility index; *TAPSE* tricuspid annular plane systolic excursion; *LVOT* left ventricular outflow tract; *VTI* velocity time integral; *LVEF* left ventricular ejection fraction; *AKI* acute kidney injury; *ICU* intensive care unit; *MV* mechanical ventilation; *KDIGO* Kidney Disease Improving Global Outcomes; *RRT* renal replacement therapy

* CVP missing for 24 patients (24 were in the VExUS < 2 group)

**Supplementary Table 2** Association between VExUS grades and outcomes

|  | **Generalized Estimating Equation Model** | | | | | | |
| --- | --- | --- | --- | --- | --- | --- | --- |
|  | **Unadjusted OR (95% CI)** | | | **Adjusted* OR (95% CI)** | | | |
|  | **OR** | **95% CI** | ***p*-value** |  | **OR** | **95% CI** | ***p*-value** |
| **AKI** | | | | | | | |
| VExUS 0 | Ref |  |  | Ref | |  |  |
| VExUS 1 | 1.69 | 0.72–3.98 | 0.228 | 1.68 | | 0.7–4.02 | 0.244 |
| VExUS 2 | 3.63 | 1.03–12.71 | 0.044 | 3.11 | | 0.83–11.62 | 0.092 |
| VExUS 3 | 0.6 | 0.05–7.08 | 0.688 | 0.49 | | 0.07–3.21 | 0.453 |
| **30-day mortality** | | | | | | | |
| VExUS 0 | Ref |  |  | Ref | |  |  |
| VExUS 1 | 0.47 | 0.17–1.28 | 0.139 | 0.51 | | 0.18–1.44 | 0.203 |
| VExUS 2 | 0.88 | 0.27–2.94 | 0.84 | 0.76 | | 0.24–2.42 | 0.639 |
| **ICU mortality** | | | | | | | |
| VExUS 0 | Ref |  |  | Ref | |  |  |
| VExUS 1 | 0.4 | 0.16–1.05 | 0.062 | 0.37 | | 0.13–1.07 | 0.066 |
| VExUS 2 | 1.1 | 0.35–3.39 | 0.874 | 0.78 | | 0.25–2.43 | 0.671 |
| VExUS 3 | 0.71 | 0.06–8.26 | 0.78 | 0.74 | | 0.1–5.64 | 0.77 |
| **RRT requirement** | | | | | | | |
| VExUS 0 | Ref |  |  | Ref | |  |  |
| VExUS 1 | 1.18 | 0.4–3.52 | 0.767 | 1.03 | | 0.34–3.1 | 0.96 |
| VExUS 2 | 2.93 | 0.85–10.14 | 0.089 | 2.45 | | 0.63–9.49 | 0.197 |
| VExUS 3 | 2.44 | 0.2–29.94 | 0.484 | 1.61 | | 0.29–8.87 | 0.585 |

*OR* odds ratio; *CI* confidence interval; *AKI* acute kidney injury; *VExUS* Venous Excess Ultrasound; *ICU* intensive care unit; *RRT* renal replacement therapy

* adjusted for age, sex, CKD and APACHE Ⅱ score

**Supplementary Table 3** Logistic regression results with AKI as dependent variable and VExUS at different timepoints as covariates

|  | **OR** | **95% CI** | ***p*-value** |
| --- | --- | --- | --- |
| **VExUS at day 1 (n = 108)** |  |  | 0.144 |
| VExUS 0 | Ref | Ref | Ref |
| VExUS 1 | 1.69 | 0.72–4.03 | 0.228 |
| VExUS 2 | 3.63 | 1.1–14.32 | 0.044 |
| VExUS 3 | 0.6 | 0.03–6.68 | 0.688 |
| **VExUS at day 3 (n = 104)** |  |  | 0.121 |
| VExUS 0 | Ref | Ref | Ref |
| VExUS 1 | 0.6 | 0.26–1.39 | 0.238 |
| VExUS 2 | 1.83 | 0.52–7.51 | 0.363 |
| VExUS 3 | 4.69 ×10^06 | 0–inf | 0.988 |
| **VExUS at day 5 (n = 95)** |  |  | 0.406 |
| VExUS 0 | Ref | Ref | Ref |
| VExUS 1 | 0.74 | 0.3–1.78 | 0.495 |
| VExUS 2 | 2.94 | 0.4–59.42 | 0.348 |
| VExUS 3 | 4.23 ×10^06 | 0–inf | 0.992 |
| **VExUS at day 1 (n = 108)** |  |  |  |
| VExUS < 2 | Ref | Ref | Ref |
| VExUS ≥ 2 | 2.12 | 0.76–6.49 | 0.162 |
| **VExUS at day 3 (n = 104)** |  |  |  |
| VExUS < 2 | Ref | Ref | Ref |
| VExUS ≥ 2 | 2.81 | 0.89–10.76 | 0.096 |
| **VExUS at day 5 (n = 95)** |  |  |  |
| VExUS < 2 | Ref | Ref | Ref |
| VExUS ≥ 2 | 4.08 | 0.62–79.9 | 0.208 |

*OR* odds ratio; *CI* confidence interval; *VExUS* Venous Excess Ultrasound

**Supplementary Table 4** Association between VExUS ≥ 2 and clinical outcomes in the septic shock subgroup

| **Outcomes** | **Generalized Estimating Equation Model** | | | | | | |
| --- | --- | --- | --- | --- | --- | --- | --- |
|  | **Unadjusted OR (95% CI)** | | | **Adjusted* OR (95% CI)** | | | |
|  | **OR** | **95% CI** | ***p*-value** |  | **OR** | **95% CI** | ***p*-value** |
| AKI | 2 | 0.62–6.49 | 0.248 | 1.87 | | 0.61–5.8 | 0.275 |
| 30-day mortality | 0.62 | 0.16–6.44 | 0.493 | 0.6 | | 0.16–2.21 | 0.439 |
| ICU mortality | 1.43 | 0.45–4.54 | 0.545 | 1.22 | | 0.38–3.9 | 0.736 |
| Requirement for RRT | 2.04 | 0.59–7 | 0.258 | 2.19 | | 0.52–9.18 | 0.284 |

*OR* odds ratio; *CI* confidence interval; *AKI* acute kidney injury; *ICU* intensive care unit; *RRT* renal replacement therapy

*adjusted for age, sex, CKD and APACHE Ⅱ score. The reference level was VExUS < 2.


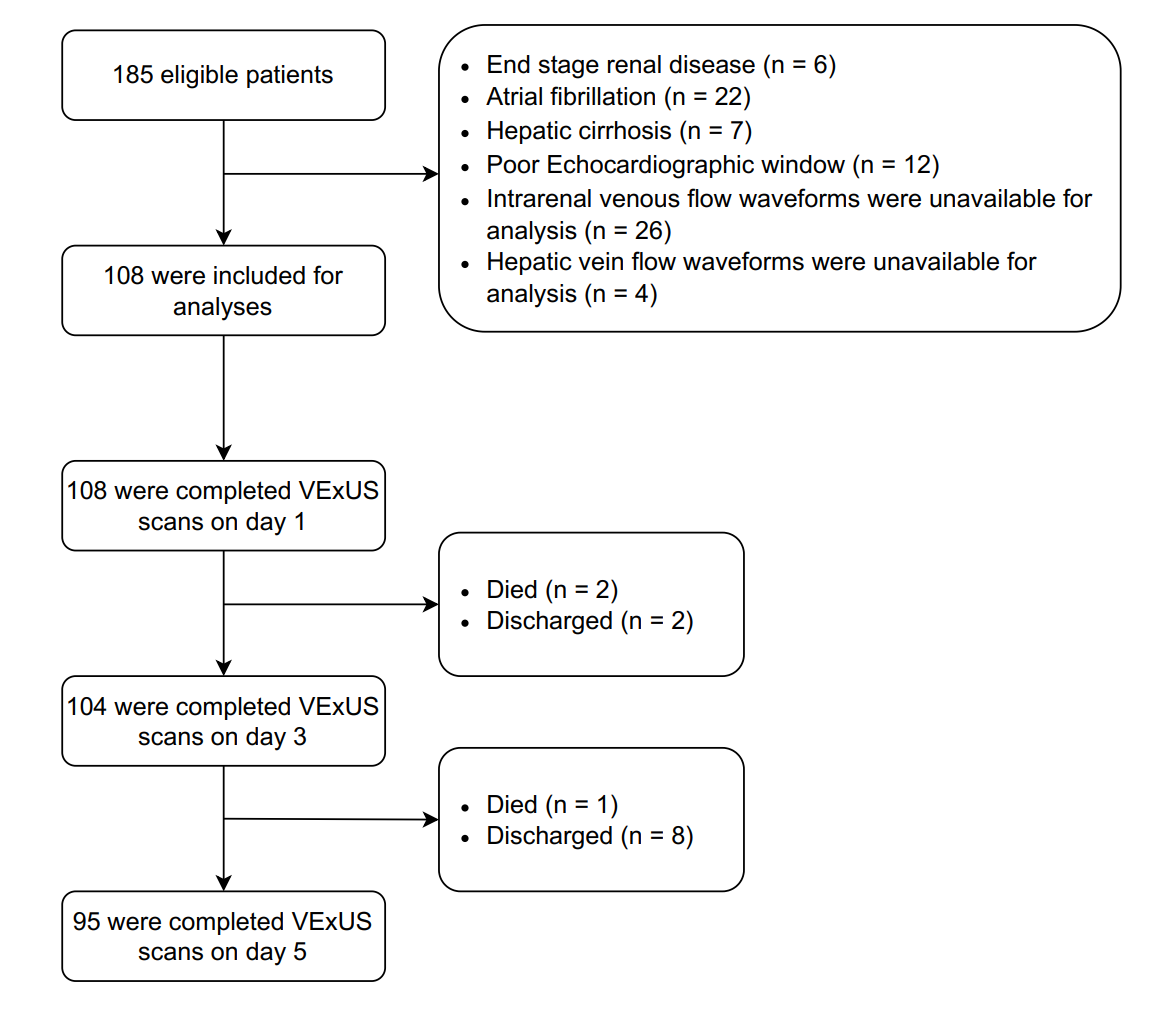


**Supplementary Figure 1** Study flow chart. VExUS denotes Venous Excess Ultrasound
